# US HOSPITAL INFRASTRUCTURE: ARE ALZHEIMER’S CENTERS PREPARED TO ADDRESS DEMENTIA NATIONWIDE?

**DOI:** 10.1093/geroni/igad104.2902

**Published:** 2023-12-21

**Authors:** Angela Gutierrez, Cory Cronin, Berkeley Franz, Graciela Muniz-Terrera

**Affiliations:** Ohio University, Athens, Ohio, United States; Ohio University, Athens, Ohio, United States; Ohio University Heritage College of Osteopathic Medicine, Athens, Ohio, United States; Ohio University, Athens, Ohio, United States

## Abstract

Evidence on the U.S. hospital infrastructure to address dementia is scant, obscuring health improvement efforts. This study investigates the availability of Alzheimer’s Centers (ACs) in U.S. hospitals. Data come from the: (1) 2010 – 2021 American Hospital Association Annual Survey; (2) 2020 Area Health Resource File; and (3) 2020 U.S. Census. Utilizing data from U.S. general hospitals (n = 3,251), we employed multivariable logistic regression to examine hospital (size, ownership, teaching status), county (metropolitan, poverty, racial/ethnic county composition), and regional (U.S. region) predictors of AC availability (Yes/No). ACs in U.S. hospitals increased from 4.5% in 2010 to 5.9% in 2020. Large hospitals (>399 beds) had approximately 14 times higher odds of having an AC than small hospitals (< 50 beds; OR = 14.0; 95% CI = 6.44 – 30.46). Counties with a higher proportion of Latinx residents, relative to non-Latinx Whites, had lower odds of having an AC (OR = 0.05; 95% CI = 0.01 – 0.41). Northeastern (OR = 1.92; 95% CI = 1.15 – 3.22) and Midwestern (OR = 2.12; 95% CI = 1.34 – 3.37) hospitals had higher odds of having an AC than Southern hospitals. Findings extend our understanding of U.S. hospital capacity to address dementia. Although ACs have increased over time, hospitals in non-metropolitan and Southern counties, and those in counties with a high proportion of Latinx adults are less likely to have an AC. To address dementia needs fully, investment in a national infrastructure is critical.

